# Editorial: Evolution and Biodiversity of Wild Polyploids

**DOI:** 10.3389/fpls.2021.723439

**Published:** 2021-07-20

**Authors:** Elvira Hörandl, Christoph Oberprieler, Karol Marhold, Natascha D. Wagner

**Affiliations:** ^1^Department of Systematics, Biodiversity and Evolution of Plants, University of Göttingen, Göttingen, Germany; ^2^Institute of Plant Sciences, Evolutionary and Systematic Botany, University of Regensburg, Regensburg, Germany; ^3^Institute of Botany, Plant Science and Biodiversity Centre, Slovak Academy of Sciences, Bratislava, Slovakia; ^4^Department of Botany, Faculty of Science, Charles University, Prague, Czechia

**Keywords:** polyploidy, evolution, biogeography, biodiversity, repeatomes, transcriptomes

Polyploidy is a major driver of evolution in plants. Angiosperms have undergone several polyploidization events in their evolutionary history (Leebens-Mack et al., 2019, Van De Peer et al., 2017) and polyploidy is an important factor causing speciation and influencing diversification of flowering plants (Abbott et al., 2013, Soltis et al., 2014b). However, the processes of evolutionary origin, establishment, speciation and post-origin evolution of polyploid plants are still not well-understood. Furthermore, most research on polyploids is performed on few model taxa or on crop plants. Overcoming the gap between cultivated model organisms and natural systems is crucial for a complete understanding of polyploid evolution. Here we present a symposium on polyploid evolution in wild plants including 10 case studies that can be grouped around three major themes: first, origins and evolutionary histories of polyploids; second, aspects of genome evolution in polyploids; and third, the relationships between polyploidy and biogeography and ecology.

Natural origins of polyploid species, their phylogenetic relationships, and the impact of polyploidy on diversification are major topics of biodiversity research. Melichárková et al. demonstrate multiple and recurrent origins of autopolyploids and inter-cytotype matings in the genus *Cardamine*. This neopolyploid complex diversified further *via* allopatry and ecological differentiation. Reconstructing phylogenies of genera including polyploids have been notoriously difficult due to the frequent origin of polyploid species *via* hybridization (allopolyploidy). In the genus *Salix*, Wagner et al. elucidate multiple polyploidization events and post-origin genome evolution of mesopolyploid species by using phylogenomic approaches. Their results supported allopolyploid origins and their establishment as distinct species. Paule et al. reconstruct genome size evolution in the big plant family *Bromeliaceae* based on the phylogeny of this family. Results suggest that polyploidy and dysploidy have played a role in the survival of early diverged lineages under extreme climatic conditions, but did not enhance net diversification. Finally, Meudt et al. analyze polyploid frequencies in floras of oceanic islands and use phylogenetic path analysis to estimate effects of polyploidy on diversification. The results suggest that endemic diversification and speciation of the analyzed island floras were shaped by polyploidy in many cases. These papers seem to support also the general pattern that young polyploid complexes tend more to increase diversification rates than older ones (Landis et al., 2018).

The evolution of polyploid genomes is highly dynamic (Comai, 2005; Chen, 2007), but many molecular consequences of whole genome duplication are still not well-understood. Zagorski et al. analyze the repeatome of *Hieracium* species and their synthetic F1 hybrids by NGS analyses and FISH. They find a marked influence of hybridization on satellite and rDNA dynamics, whereas transposable elements showed no significant bursts but rather dosage-dependent patterns. Analyses of the repeatome in the mesopolyploids of *Heliophila* by Dogan et al. reveal a highly dynamic evolution of retrotransposons and tandem repeats, reflecting the post-origin cladogenesis in the genus. Boatwright et al. analyze gene expression patterns of homoeologs in mesopolyploid populations of *Tragopogon* compared to their parental species and reveal similar and convergent expression changes, but also stochastic silencing of loci. Moreover, the authors found significantly more non-additive expression in mesopolyploids compared to recently formed neopolyploids.

Polyploids are thought to have advantages for ecological niche shifts (Marchant et al., 2016, Soltis et al., 2014b) and for range expansions, specifically to more northern regions (Rice et al., 2019). Buono et al. conducted phylogeographical analyses, niche modeling and crossing experiments on two European polyploid *Veronica* species and found that despite some introgression species boundaries are maintained by spatial and ecological isolation. Duchoslav et al. analyze the niche dynamics of various cytotypes of *Allium oleraceum* and find niche expansions in tetraploids, but not in higher ploidy levels. High ploidies rather showed unfilled niches and a tendency toward synanthropic habitats. Spoelhof et al. use a modeling approach to understand the establishment of polyploids in natural habitats by including spatial factors and relationships between organisms, habitat shape, and population density. Results suggest that narrow, constrained habitats such as roadsides and coastlines may enhance polyploid establishment.

The symposium highlights the diversity of evolutionary processes that are connected with polyploidy. Several papers point at the importance of post-origin evolution and diversification of polyploids, a topic that is hitherto been neglected in biodiversity research. We are at the infancies of understanding genome evolution and functionality in natural polyploid model systems. Here we present insights into repeatome evolution and gene expression studies. Whole genome sequencing and building reference genomes for wild model systems are highly needed to understand the consequences of polyploidization on genome structure and function. The symposium further shows various biogeographical and ecological scenarios for polyploids. Altogether, the case studies in this symposium refute the idea that polyploidization would be a dead end of evolution, as proposed by some researchers based on simple diversification statistics (Mayrose et al., 2011). The bias in such studies by undersampling, by neglecting reticulate evolution and different ages of diploids vs. polyploids was reviewed before (Soltis et al., 2014a). Careful case studies as presented here will provide a better understanding of the realized ecological and evolutionary potentials of polyploids.

## Author Contributions

EH wrote the Editorial with contributions from all co-authors. All authors contributed to the article and approved the submitted version.

## Conflict of Interest

The authors declare that the research was conducted in the absence of any commercial or financial relationships that could be construed as a potential conflict of interest.
